# Up‐to‐dateness of German patient versions of clinical practice guidelines and potential influencing factors: A mixed‐methods study

**DOI:** 10.1002/gin2.70017

**Published:** 2025-02-03

**Authors:** Irma Hellbrecht, Nadja Könsgen, Dawid Pieper, Barbara Prediger, Jessica Breuing

**Affiliations:** ^1^ Institute for Research in Operative Medicine (IFOM) Witten/Herdecke University Cologne Germany; ^2^ Institute for Health Economics and Clinical Epidemiology University of Cologne Cologne Germany; ^3^ Faculty of Health Sciences Brandenburg Brandenburg Medical School (Theodor Fontane), Institute for Health Services and Health System Research Rüdersdorf Germany; ^4^ Center for Health Services Research, Brandenburg Medical School (Theodor Fontane) Rüdersdorf Germany

**Keywords:** evidence‐based patient information, expert interviews, influence factors, patient guideline, patient versions of clinical practice guidelines, updating, up‐to‐dateness

## Abstract

**Introduction:**

Clinical practice guidelines (CPGs) provide recommendations and are a fundamental part of clinical practice. Many guideline organisations also produce patient versions of CPGs (PVGs). To explore the up‐to‐dateness of German PVGs, potential methodological influence factors and experts' perspectives, we applied a convergent mixed‐methods design.

**Methods:**

First, a literature search to identify PVGs was performed between October 2022 and January 2023. We searched the websites of German guideline organisations, Google and the reference lists of included PVGs. We screened the title, downloaded the documents if relevant and retrieved the underlying CPGs and methods reports of CPGs. We aggregated the literature search dates of CPGs and calculated the time between the CPG literature search and PVG publication. Second, interviews with experts in the PVG development were conducted and analysed using qualitative content analysis (Mayring) with MAXQDA.

**Results:**

49 PVGs were included and analysed. A median of 36 months elapsed between the literature search of CPGs and the publication of PVGs. A median of 25 months passed between the literature search and publication of CPGs, and a median of 7.5 months elapsed between the CPG and PVG publication. Six interviews were conducted and interviewees mostly perceived PVGs as up‐to‐date. However, they identified exceptions in the up‐to‐dateness depending on the topic or thematic chapters of PVGs. Interviewees mentioned different influencing factors such as the scientific progress and the editorial process of PVGs.

**Discussion:**

Our findings underline potential issues with the up‐to‐dateness of PVGs. In the context of a fast‐moving evidence basis, it seems doubtful whether PVGs actually reflect the current state of knowledge, especially in fields with high research activity. However, some factors may not be modifiable because they essentially contribute to the quality assurance of PVGs. Further research is desirable to investigate possible measures to improve the up‐to‐dateness of PVGs.

## Key points

Key findings

- The median time lag between the literature search of clinical practice guidelines (CPGs) and the publication of patient versions of guidelines (PVGs) was 36 months, with significant variability (range: 7–83 months).
- Expert interviews revealed that PVGs are generally perceived as up‐to‐date, but challenges exist in fast‐evolving fields like oncology.
- Factors contributing to delays include editorial processes, print version availability, and glossary integration.

What this adds to what was known?

- Timeliness is essential for PVGs to maintain their relevance and accuracy for patients, yet the development and publication processes can face structural inefficiencies.
- Current practices during the guideline development stage significantly influence the overall timelines for PVG production.
- There is a need for integrated workflows between CPG and PVG production to enhance synchronicity and efficiency.

What is the implication and what should change now?

- Initiating the PVG development during the early stages of CPG creation can minimize delays and ensure up‐to‐date recommendations for patients.
- The adoption of digital tools and transitioning towards ‘living guidelines’ could streamline updates and revisions.
- Improved resources, enhanced collaboration between stakeholders, and structured timelines are essential to expedite PVG production.

## 1 INTRODUCTION

Clinical practice guidelines (CPGs) provide clear recommendations^1^ and aim to represent the current state of evidence; therefore, it is crucial to keep them up‐to‐date.^2^ However, there is controversy about the frequency and process of updating CPGs,^3^ especially in the context of a rapidly evolving evidence base.^4^ According to Shekelle et al. more than three‐quarters of CPGs of the US Agency for Healthcare Research and Quality published between 1990 and 1996 were outdated by 2001.^5^ Previous work also showed that 20% of recommendations become outdated within 3 years.^6^

According to van der Weijden et al., a PVG is an ‘explanation of a specific condition or (health) care issue based on a guideline; made available to patients and their next of kin; provides information on available care choices and the care that they can expect from the care process’.^7^ PVGs reflect the recommendations of CPGs in plain language^8^ and have the goal to empower patients, foster their self‐responsibility and promote their understanding of diseases.^9^, ^10^ In Germany, CPGs and PVGs are mainly developed by the National Disease Management Guidelines program (German: NVL), German Guideline Program in Oncology (GGPO) and Association of the Scientific Medical Societies (German: AWMF). Within the NVL and GGPO guideline programs the development of PVGs is mandatory.^11^ Since a lack of up‐to‐dateness can limit patients' trust in PVGs,^12^ they should be updated in a timely manner.^8^ No data on the up‐to‐dateness of PVGs is available so far. We explored the up‐to‐dateness of PVGs in Germany, the potential methodological influence factors and experts' perspectives. The aim of this study was to complement quantitative results on the up‐to‐dateness of PVGs with qualitative expert interviews in a convergent mixed‐methods design.

## 2 METHODS

### 2.1 Study design

A systematic search was conducted to identify PVGs in Germany and to analyse their up‐to‐dateness. We explored the time between CPG literature search and PVG publication to analyse the up‐to‐dateness of PVGs. To extend the breadth of investigation, we conducted interviews with experts in the PVG development. The purpose of the interviews was to gain deeper knowledge about the development process and to explore potential methodological influence factors and possibilities for improvement. We considered elements that either constitute the development process or are specific to the PVG (e.g., number of pages) as methodological influence factors. Both study components were analysed independently and aggregated during the overall interpretation. The purpose of applying a convergent mixed‐methods design was to enhance the findings from both methods. An unpublished a‐priori developed proposal for the Master's Thesis on which this study was based served as a study protocol (see Additional file 1). There was no funding for this study.

To report our results, we used the PRISMA Statement^13^ and the consolidated criteria for reporting qualitative research (COREQ), where applicable.^14^

### 2.2 Systematic search

#### 2.2.1 Eligibility criteria

Eligible PVGs had to meet the following criteria: published in German, freely accessible, explicit link to corresponding CPG (including version), final and most recent version, at least month and year of the PVG publication date, and date of the research strategy of CPG available. In our understanding, a PVG is a comprehensive document reflecting the content of a CPG. Accordingly, patient information consisting of 1–2 pages was not deemed relevant. For instance, the German Agency for Quality in Medicine produces such brief patient information that does not meet our understanding of a PVG.^15^ Due to varying terminology,^16^, ^17^ we generally considered different designations such as ‘patient information’ or ‘patient version’ to be relevant, although we had prospectively planned to include only documents that were defined as PVGs. Authors were contacted if the PVG publication date or the version of the underlying CPG or CPG report were not evident. We excluded living PVGs due to difficulties in comparison. In some cases, PVGs were published earlier than the CPGs. Therefore, we defined a threshold and excluded PVGs, which were published more than 6 months earlier than the CPGs.

#### 2.2.2 Information sources and search strategy

We conducted a systematic search between October and November 2022 to identify national PVGs. The websites of the German guideline organisations NVL, GGPO and AWMF were searched. Moreover, we screened Google and the reference lists of included PVGs in January 2023. On the websites of the NVL and GGPO, we selected all available tabs related to PVGs. We selected the document type ‘Patientenleitlinie’ on the website of the AWMF and retrieved Google by the German keyword ‘Patientenleitlinie’ to identify PVGs. The selection process was carried out in three subsequent steps. Firstly, documents were screened by title by one author (IH). Secondly, documents deemed relevant were downloaded and screened. The underlying CPGs and methods reports of CPGs were retrieved in the third step. Finally, the reference lists of included PVGs were screened. The second and third screening steps were performed by two independent authors (IH, NK). We resolved disagreements by discussion or by involvement of a third author (JB).

#### 2.2.3 Data extraction

While piloting a Microsoft Excel extraction form, reviewer IH extracted data from a PVG and verified it with reviewer NK. Subsequently, the form was adapted. The first reviewer (IH) performed data extraction and the second reviewer (NK) verified it. Disagreements were discussed until consensus was reached or by consulting a third reviewer (JB). We extracted general characteristics of PVGs, the CPG and PVG publication dates and the CPG literature search dates (see Table 1 for further details). In addition, we extracted data on the characteristics of PVGs (such as number of pages or availability of PVGs as print version [y/n], of a glossary [y/n] and of figures and/or tables [y/n]). We suspected that these factors, which contribute to the quality of a PVG, would extend the time required for the PVG development.

**Table 1:** Data extracted from PVGs and CPGs.^a^

General data	Description
Publisher	Organisation that published the PVG
Medical condition	Health issue/topic addressed by the PVG, then classified into categories according to ICD

Up‐to‐dateness	Description
PVG publication date CPG publication date CPG literature search dates	At least month and year. If a time span (e.g., March 2017) was reported, the median of that period was extracted A) Dates of manual searches and searches of previous versions of CPGs were not extracted, due to a lack of resources and our aim to depict the most current literature search, respectively B) Dates were extracted separately for each search strategy and database. E.g.: *Both Medline and Cochrane Library were searched on 15.01.2013 to answer a PICO question* → We extracted the date twice, since two different databases were searched at this time

Abbreviations: CPG, clinical practice guideline; ICD, international classification of diseases; PVG, patient version of CPG.

^a^ This table shows the extracted data for which a more detailed explanation was necessary. All other data we have extracted is described in the text.

We extracted the CPG literature search dates according to the following approach: (I) in case of de novo searches and searches for existing systematic reviews and meta‐analyses, we only extracted the dates of literature searches carried out in databases enabling a systematic search such as PubMed. (II) Mostly, there were no systematic searches in case of guideline adaptions. Therefore, we extracted all available search dates including literature searches conducted on websites of organisations and in web libraries of institutions.

#### 2.2.4 Data analysis

**Figure 1:**
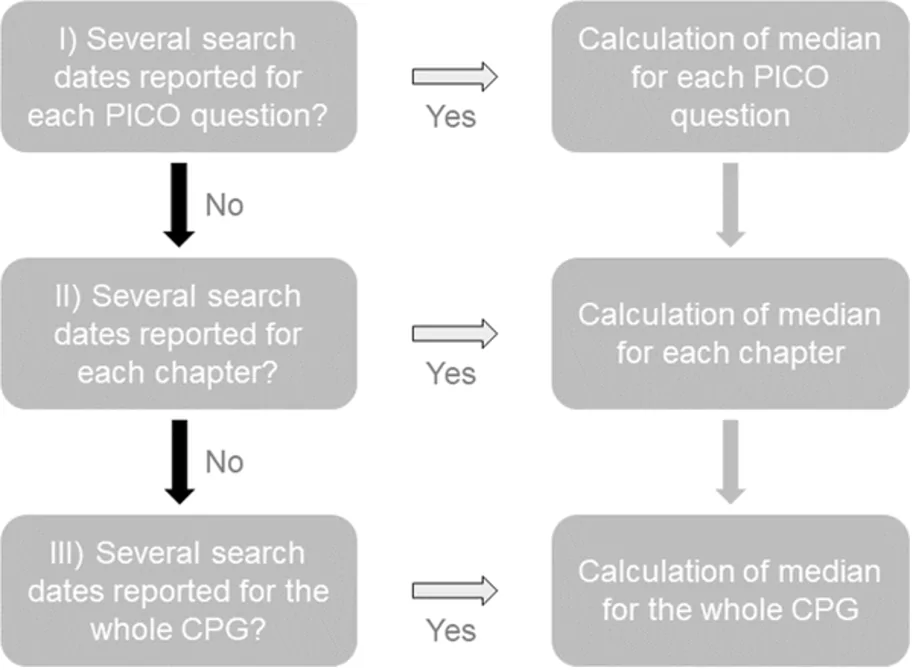
Algorithm of the aggregation of literature search dates.

We aggregated literature search dates using the algorithm in Figure 1 to calculate one search date per CPG and determine the time between CPG literature search and PVG publication. Additionally, we also calculated the time from the literature search to CPG publication and the time between CPG and PVG publication. The median (IQR) and mean (SD) times for these periods were reported across all PVGs and CPGs. To analyse the relationship between PVG up‐to‐dateness and PVG characteristics, we calculated the median up‐to‐dateness for PVGs with and without certain characteristics, without performing statistical tests for group differences.

### 2.3 Qualitative interviews

#### 2.3.1 Recruitment

We arbitrarily contacted 30 authors of PVGs via email with the aim of recruiting at least five. Due to limited resources in the context of the Master's Thesis, the sample size was rather small. The invitation included information on the aim of the study, the participation information and privacy statement. Participants gave written informed consent for participation.

#### 2.3.2 Data collection

One author (IH), a female student at the University of Cologne and researcher at the Witten/Herdecke University, conducted semi‐structured interviews via telephone. She had no contact with the participants before the interviews, apart from the invitation and scheduling of appointments. Except for one case (a former colleague of the interviewer), there was no relationship between the participants and the interviewer. Participants received no incentives. One author (IH) prepared the interview guide before the interviews and in consultation with two other authors (NK, JB). The guide comprised of three main sections: (1) general information about the participant, (2) development process of PVGs, and (3) up‐to‐dateness of PVGs. No pilot testing of the interview guide was conducted, but the first interview was used to reveal potentially necessary adjustments. The interview guide was developed on the basis of the literature search (see Additional file 2). All interviews, were conducted from home between January and February 2023 and audio recorded with no field notes or repeat sessions. Participants chose their own settings.

#### 2.3.3 Data processing and analysis

An external institution conducted the verbatim transcription of the interviews. The interviewer (IH) checked the final transcripts. Participants did not provide feedback on the results. MAXQDA software was used to analyse the transcribed interviews according to the qualitative content analysis.^18^ Before the interviews, one reviewer (IH) deductively defined the main categories (codes) based on the core themes of the interview guide. Afterwards, she refined the category system by inductively adding subcategories (subcodes) during the analysis of the material. Due to the context of a Master's Thesis, only one author (IH) analysed the interviews in consultation with two other authors (NK, JB). For data coding system, including rules of coding and specifications defined for each code see Additional file 3.

## 3 RESULTS

### 3.1 Systematic search

#### 3.1.1 Selection

**Figure 2:**
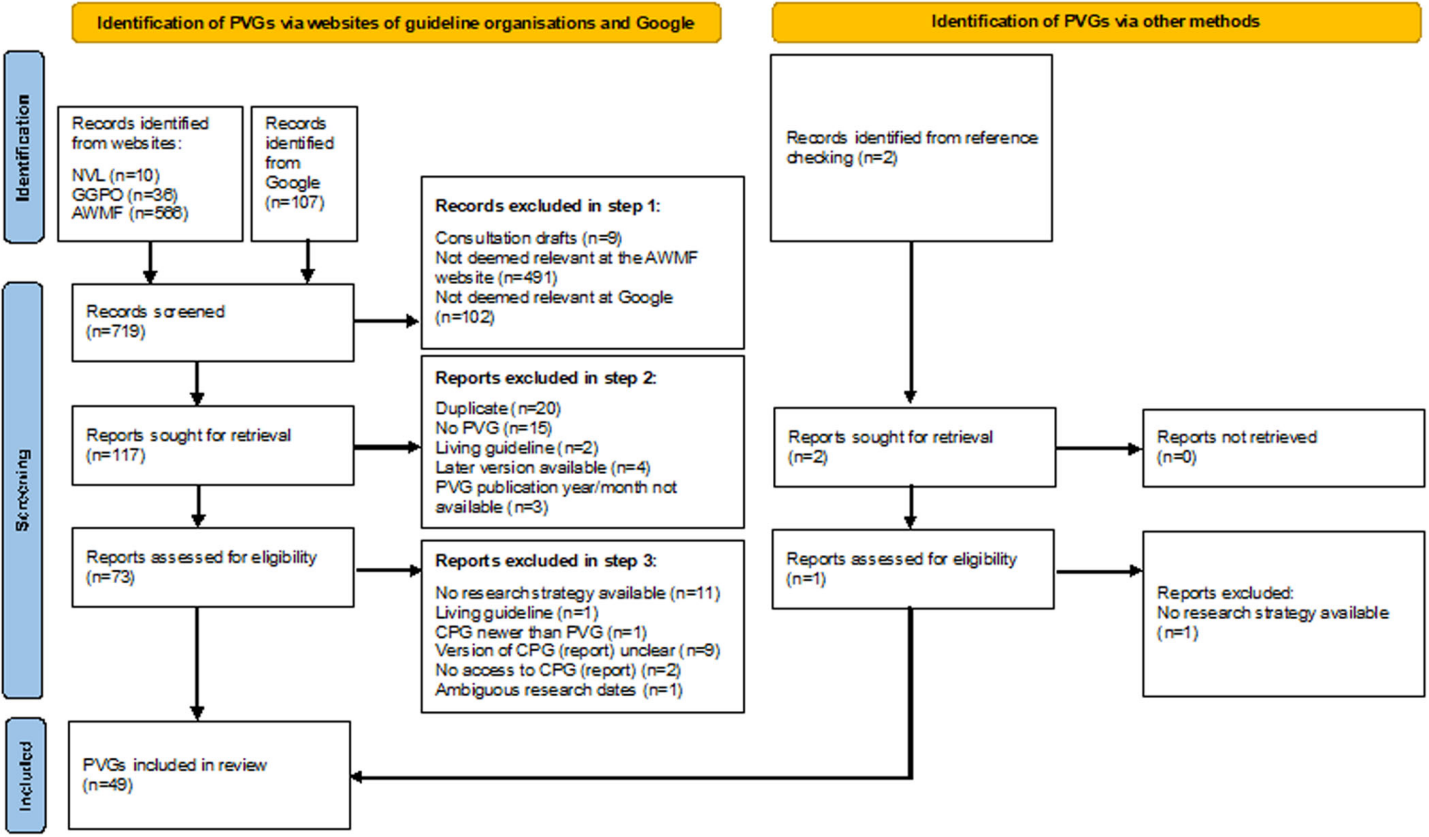
Flow diagram of included PVGs. Based on the preferred reporting items for systematic reviews and meta‐analyses (PRISMA) flow chart.^14^ AWMF, Association of the Scientific Medical Societies in Germany; CPG, Clinical practice guideline; GGPO, German Guideline Program in Oncology c/o German Cancer Society; NVL, National Disease Management Guidelines Program; PVG, patient version of CPG.

The search on the websites of the German guideline organisations and Google yielded 719 documents, of which 671 were excluded. Reference checking of all 48 included PVGs yielded two potentially relevant PVGs. Finally, we included 49 PVGs.^19^, ^20^, ^21^, ^22^, ^23^, ^24^, ^25^, ^26^, ^27^, ^28^, ^29^, ^30^, ^31^, ^32^, ^33^, ^34^, ^35^, ^36^, ^37^, ^38^, ^39^, ^40^, ^41^, ^42^, ^43^, ^44^, ^45^, ^46^, ^47^, ^48^, ^49^, ^50^, ^51^, ^52^, ^53^, ^54^, ^55^, ^56^, ^57^, ^58^, ^59^, ^60^, ^61^, ^62^, ^63^, ^64^, ^65^, ^66^, ^67^ The selection process is illustrated in Figure 2 and the excluded items can be found in Additional file 4.

#### 3.1.2 Characteristics

The PVGs were published between 2012 and 2022, had a mean number of 88 pages, and addressed various medical indications according to the ICD‐10 classification system (see Table 2). The involvement of patients into the development process, the use of figures and/or tables, and the provision of a glossary were common. Mostly, PVGs contained an introduction to or highlighting of keywords for the grade of recommendation. The grade of recommendation and the wording from the CPGs were also used in the PVGs. Only about one‐third of the PVGs went through a consultation phase and only one‐third were available as print versions. See Table 3 for further details on the key characteristics of PVGs.

**Table 2:** Topics of PVGs.

Medical indications (according to ICD‐10 catalogue)	Percentage of PVGs (n)
Neoplasms	33 (16)
Endocrine, nutritional or metabolic diseases	16 (8)
Mental and behavioural disorders	12 (6)
Diseases of the musculoskeletal system or connective tissue	10 (5)
Diseases of the nervous system	6 (3)
Diseases of the respiratory system	4 (2)
Diseases of the circulatory system	4 (2)
Chronic pain^a^	4 (2)
Diseases of the digestive system	4 (2)
Diseases of the ear and mastoid process	2 (1)
Conditions related to sexual health^b^	2 (1)
Pregnancy, childbirth or the puerperium	2 (1)

Abbreviations: ICD, international classification of diseases; n, number; PVG, patient version of a clinical practice guideline.

^a^ Different from ICD system. Two PVGs were classified under the term ‘chronic pain’ because their assignment to the ICD‐10 classification system was unclear.^42^, ^57^

^b^ ICD‐11, since definition of ICD‐10 was perceived as discriminatory.

**Table 3:** Key characteristics of PVGs.

Characteristics of PVGs	Percentage of PVGs (n)
Patient involvement	84 (41)
Use of figures/tables	82 (40)
GoR introduced or keywords highlighted	80 (39)
Use of glossary	76 (37)
Addresses to support groups	71 (35)
PVG available as print	35 (17)
Public consultation phase	33 (16)
Separate PVG report available	25 (12)

*Note*: Although prospectively planned, we did not extract information on the starting time of the PVG development. We found that the extraction of the starting time was very subjective. Therefore, we inquired that information during the expert interviews.

Abbreviations: GoR, Grade of recommendation; n, number; PVG, Patient version of a clinical practice guideline.

#### 3.1.3 Up‐to‐dateness

**Figure 3:**
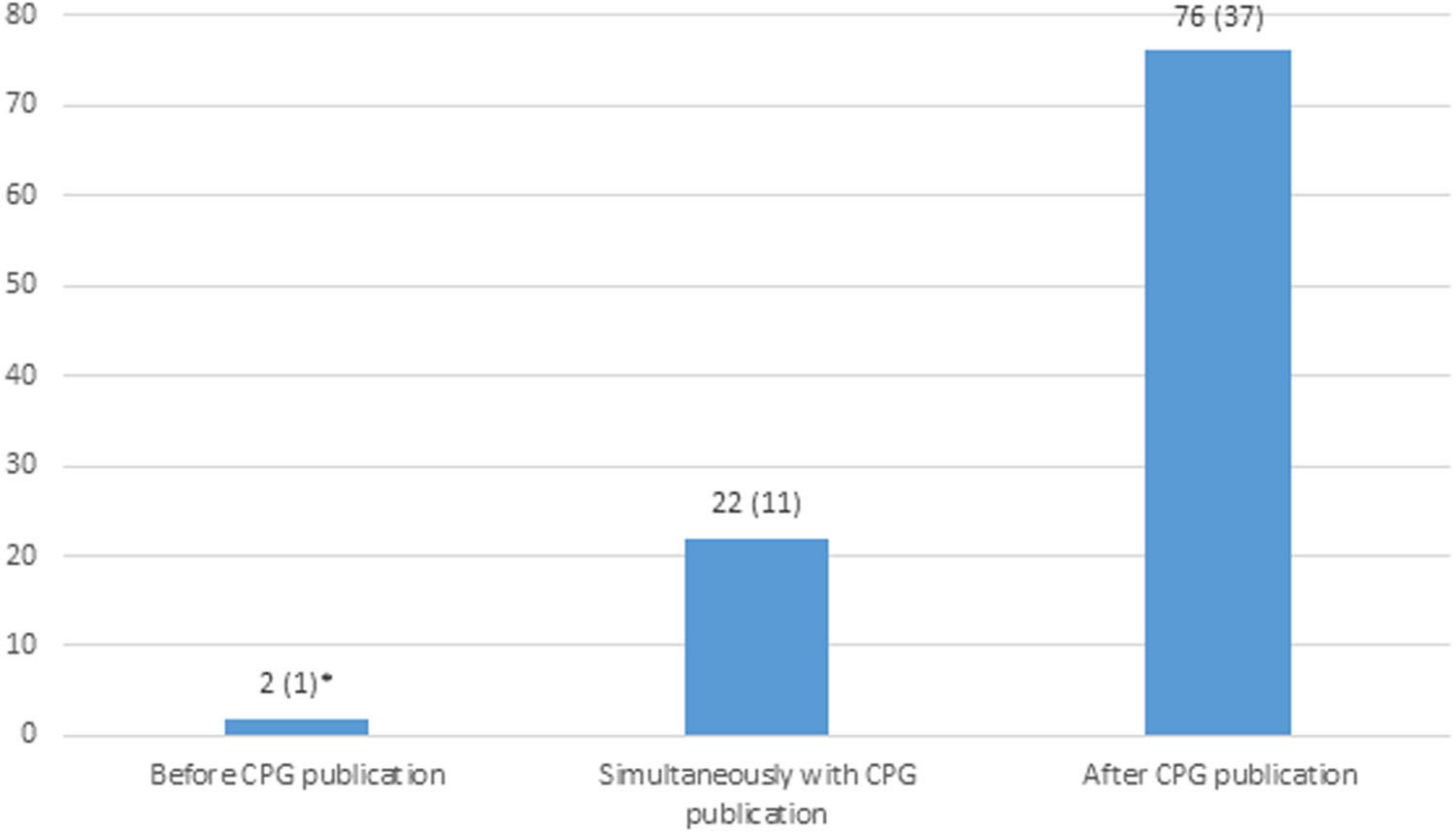
Time of PVG publication. Percentage of PVGs (n). CPG, Clinical practice guideline; PVG, patient version of CPG; *PVG was published <6 months before the CPG.

The median time from the CPG literature search to PVG publication was 36 months (range 7–83). Between the literature search and CPG publication, the median time was 25 months (range 6–56). PVGs were published a median of 7.5 months (range 0–57) after the CPG (see Table 4). One PVG was excluded from the calculation as it was published before the CPG, resulting in a negative time period. For more details, see Figure 3 and Table 5.

**Table 4:** Up‐to‐dateness.

Time period (months)	Median	IQR	Mean	SD
CPG literature search – CPG publication	25	17	25.24	11.62
CPG publication – PVG publication	7.5	12.25	10.71	11.69
CPG literature search – PVG publication	36	19	35.78	17.53

Abbreviations: CPG, clinical practice guideline; IQR, interquartile range; PVG, patient version of CPG; SD, Standard deviation.

**Table 5:** Up‐to‐dateness of PVGs in relation to characteristics.

	Median up‐to‐dateness of PVGs in months (n)	
Characteristics of PVGs	Characteristic fulfilled	Characteristic not fulfilled
Addresses to support groups	38 (35)	20.5 (14)
Figures and/or tables	36.5 (40)	28 (9)
Glossary	37 (37)	21 (12)
Introduction to or highlighting of keywords for the GoR	37 (39)	22.5 (10)
Print version	41 (17)	27.5 (32)
Public consultation	39 (16)	21 (3)
Separate PVG methods report	35.5 (12)	36 (37)

Abbreviations: GoR, grade of recommendation; n, number; PVG, patient version of clinical practice guideline.

### 3.2 Qualitative interviews

#### 3.2.1 Main characteristics

The mean duration of the six conducted interviews was 31 min (range 23–38). Participants were mainly female and had a median age of 47 years (range 31–62). The characteristics of interview participants are shown in Table 6. The interview statements are shown in additional file 5.

**Table 6:** Characteristics of interview participants.

Interview participants	
Age (y): median (IQR)	47 (6.5)
Gender: *n* (%)	
− Female	5 (83)
− Male	1 (17)
Professional position: *n* (%)	
− Methodological	5 (83)
− Clinical	1 (17)
Years in professional position: median (IQR)	8 (9.6)
Tasks in PVG development: *n* (%)	
− Coordination of PVG development	3 (50)
− Drafting of PVG	5 (83)
− Review of PVG	1 (17)
− Dissemination	2 (33)
− Organisation of patient involvement	2 (33)
− Development of general methodology/template	1 (17)
− Drafting of PVG methods report	1 (17)

Abbreviations: IQR, interquartile range; n, number; PVG, patient version of clinical practice guideline; y, years.

#### 3.2.2 Development process of PVGs

Most participants stated that the development process usually begins during the CPG consultation phase. However, the start is also conceivable after the CPG finalisation. Participants described a multidisciplinary editorial board that discusses the procedures, goals and proposed structure of the PVG during a first kick‐off‐meeting and reviews the PVG draft version in several rounds. Participants emphasised the importance of considering patient interests during the development process of the PVG draft version. Other interested people, such as representatives of self‐help organisations, may comment during the public consultation phase. Usually, the final PVG is published on an online platform and made available to the public as PDF version.

Participants mentioned the extent and quality of the underlying CPGs as possible influence factors on the development process. One participant claimed that high‐quality background texts and evidence tables of the CPG may be beneficial to explain and justify recommendations in the PVG.

Another participant argued that members might reach their limit of capacity, if there are no changes in the composition of the editorial board. An invariable pool of members might therefore impede the development of PVGs. Finally, some participants suggested that the review process might lead to significant time lags and hinder the development process.

#### 3.2.3 Up‐to‐dateness of PVGs

Most participants perceived PVGs as up‐to‐date, although our systematic search showed, that a median of 36 months elapsed between the CPG literature search and PVG publication. However, the participants emphasised that there might be exceptions depending on the topic (fast moving topics such as cancer) and thematic chapters (e.g., chemotherapy).

Furthermore, participants mentioned time resources and professional competences of members of the editorial board as influence factors. Accordingly, motivated and trained members might promote the up‐to‐dateness of PVGs. In particular, one participant highlighted the need to train members with regard to patient information.

Another participant argued that a delayed planning of the PVG development process or a stepwise approach of CPG and PVG development might impede the up‐to‐dateness of PVGs. However, our results from the systematic search showed, that most delays occurred during the CPG development process and not during the CPG and PVG publication. Others suggested that the availability of PVGs as print versions might be time‐consuming and hindering, since the printing process could make it difficult to respond to necessary updates. Our systematic search also indicated, that there might be characteristics influencing the up‐to‐dateness of PVGs, such as the availability of a print version or glossary.

#### 3.2.4 Suggestions for improvement

Participants mentioned various suggestions for improvement. Most referred to the availability of financial resources. Others recommended building on members' time resources. One participant assumed that younger members are likely to have more time and could equally contribute to the development of PVGs.

Some participants suggested the establishment of permanent responsibilities to save time resources spend on tendering. Accordingly, the responsible members might be entrusted with the supervision of the PVG in the long term. Participants also recommended assigning more responsibilities and providing training as well as compensations to patient representatives.

Others suggested an earlier onset of the PVG development process through an earlier planning and assembly of the editorial board. Participants also suggested starting with general chapters or initiating the development process of PVGs as soon as individual chapters of the CPG are finalised. Moreover, some participants referred to the coordination of the PVG development and suggested a structured approach as well as clear definitions of responsibilities, timing and milestones.

Finally, participants recommended the digitalisation of the PVG development process and the implementation of continuously adjustable online versions according to the principle of living guidelines.

Online tools such as content management systems could accelerate the development process by providing a quick method of editing and generating content.

## 4 DISCUSSION

This convergent mixed‐methods study explored the up‐to‐dateness of PVGs in Germany and potential methodological influence factors. To our knowledge, this is the first study that has analysed the time span between the literature search of CPGs and publication of PVGs in Germany. The median time span was 36 months. In contrast, CPGs were published at a median of 25 months following the literature search and a median of 7.5 months elapsed between the CPG and PVG publication. The interview results indicated that experts in the field of the PVG development mostly perceive PVGs as up‐to‐date. Exceptions depended on the topics and thematic chapters of PVGs with a fast‐moving evidence basis.

Our findings may indicate potential issues regarding the up‐to‐dateness of PVGs, especially in light of an increasing rate of output in research.^4^ Not only is the evidence base constantly growing, but also the methodological standards for producing reviews are increasing.^4^ The median time spans investigated in this study have different implications. The longer the time between CPG literature search and CPG or PVG publication, the older the state of knowledge. The time span between CPG and PVG publication implies how quickly information is made available to patients. Assuming that a median of 36 months or even more elapses between the literature search and the publication of PVGs, information on certain topics or thematic chapters may already be outdated by that time. We found PVGs on the topics of obesity and diabetes that reflect the evidence basis of almost 7 or 6 years ago.^22^, ^24^, ^44^ According to bibliometric analyses, there is an upward trend in the number of publications in different fields of diabetes.^68^, ^69^, ^70^ Therefore, it may be considered whether these PVGs reflect the current state of knowledge and whether patients may benefit from them.

Furthermore, our results indicate that the time span between the CPG literature search and PVG publication varies widely with a minimum of 7 months^46^ and a maximum of 83 months.^44^ Our investigation indicated that the fulfillment of certain characteristics can potentially delay the publication of PVGs. Most characteristics discussed in this work substantially contribute to the quality assurance of PVGs and may not be modifiable. Therefore, the possibilities of shortening the development process without reducing the quality of PVGs might be limited. For instance, glossaries and explanations of keywords and unknown terms may foster the understanding of lay people.^71^ In fact, many research articles on PVGs emphasise the importance of readability, for example, by using lay‐friendly language.^17^ Good quality health information should also provide additional sources of support and enable users to find further information.^72^

We also found that the time between the literature search and publication of CPGs is considerably longer than the time between the CPG and PVG publication. Accordingly, delays especially occur during the CPG development process. Consequently, this may lead to an older state of knowledge within PVGs, even if the PVG development process proceeds quickly. Therefore, further research could investigate approaches to shorten the CPG development, while maintaining the methodological quality. Guideline organisations could specify criteria for the up‐to‐dateness of PVGs, define the updating process of PVGs in the methods reports or set a short updating interval for specific chapters that require frequent updating (e.g., drug therapy).

Some PVGs and CPGs were published simultaneously. The time of publication might be driven by different requirements of guideline organisations. By promoting a timely publication of CPGs and PVGs, patients may benefit from an earlier access to information. This may allow patients and healthcare professionals to make decisions based on the same level of knowledge.

The interviewees mentioned several suggestions to improve the up‐to‐dateness of PVGs. Some suggested establishing permanent responsibilities to avoid tendering and save time resources. Others argued that members might reach their limits, if the composition of the editorial board remains unchanged. Although it seems reasonable that permanent responsibilities may facilitate the collaboration of members, the involvement of new members may bring new in‐put, experiences and perspectives. Therefore, mixed teams may represent a suitable approach to improve the development and up‐to‐dateness of PVGs. Although some of the interviewees suggested an earlier onset of the development process, others discussed whether this approach might be feasible in terms of limited time resources of members. Therefore, an earlier onset could be considered, taking into account the available time resources and feasibility. Furthermore, the interviewees mentioned the digitalisation of the development process and the use of digital PVGs. However, they also emphasised the importance of print versions that still seem popular among users. Therefore, different perspectives should be considered and there is probably no single approach that will meet all requirements simultaneously.

By means of a mixed‐methods design we were able to explore the interactions of both components of this study and to obtain a differentiated understanding of PVG development and up‐to‐dateness. Although we found a median time lag of 36 months between CPG literature search and PVG publication, experts in PVG development mostly perceived PVGs as up‐to‐date. Some experts suggested an earlier onset of the PVG development to improve the up‐to‐dateness. However, our systematic search revealed that most delays occurred during the CPG development. We have found that some characteristics of PVGs could potentially delay publication, but may not be modifiable. For example, interviewees emphasized the relevance of PVG print versions, although their availability could delay the PVG publication.

Future research should consider the feasibility and value of methods to improve up‐to‐dateness, as well as patients' needs and measures for quality assurance.

### 4.1 Strength and limitations

Although this work allows a differentiated view on the up‐to‐dateness of PVGs, it has several limitations. The transparency and comprehensibility of the screening process during our systematic search might be restricted because the website of the AWMF was updated in the meantime. Moreover, we excluded PVGs if they were only available as consultation drafts at the time of the research process. We did not conduct a repetitive search due to time constraints. We excluded living PVGs, as they have a different updating frequency and were not part of our study question.

Several sticking points emerged during the extraction of CPG literature search dates. Although we did not plan to extract dates of manual searches, it may still be possible that we extracted some dates by mistake because they were not identified as manual searches. Some CPGs were reported in a way that the distinction between systematic and manual searches was not always clear. Moreover, we conducted no statistical testing to examine group differences in the context of the up‐to‐dateness and characteristics of PVGs. The comparison groups were rather small. In combination with the statistical significance test that was not carried out, it could, therefore, appear as if there were differences, even though this was not the case. The results of this investigation only provide indications of possible correlations.

Moreover, we did not perform a random selection of potential interviewees and there was a small sample size due to a limited time period for arranging interview appointments. Therefore, representativeness is limited. Most of the interviewees had a methodological background, but they differed in other characteristics. As a result, we were able to identify different perspectives in PVG development.

## 5 CONCLUSION

The median time between the CPG literature search and PVG publication was 36 months. However, most interviewees considered PVGs in Germany to be up‐to‐date. Most delays seemed to occur during the CPG development and there was variation in the time of the PVG publication. Further research should investigate the reasons for delays during the CPG development and the possibilities of a simultaneous publication of CPGs and PVGs. Guideline organisations could define the PVG updating process in the methods reports. When deriving recommendations for improving the up‐to‐dateness of PVGs, guideline organisations and patients should be involved and the methodological requirements and feasibility should be considered.

## AUTHOR CONTRIBUTIONS

Irma Hellbrecht, Nadja Könsgen, Jessica Breuing, Barbara Prediger conceived and designed the study. Irma Hellbrecht, Nadja Könsgen conducted screening and data extraction. Irma Hellbrecht, Nadja Könsgen, Jessica Breuing developed the interview guide, and Irma Hellbrecht conducted the interviews. Irma Hellbrecht developed the coding system and analysed data. Irma Hellbrecht, Nadja Könsgen, Jessica Breuing, Barbara Prediger, and Dawid Pieper drafted the manuscript. All authors reviewed drafts of the manuscript and approved the final manuscript.

## CONFLICT OF INTEREST STATEMENT

Irma Hellbrecht, Nadja Könsgen, Dawid Pieper, Barbara Prediger and Jessica Breuing are involved in the development of clinical practice guidelines. Jessica Breuing, Nadja Könsgen are involved in the development of patient version of clinical guidelines.

## ETHICS STATEMENT

This study was approved by the Witten/Herdecke University Ethical Committee (254/2022). All methods were performed in accordance with the principles of the Declaration of Helsinki. Written informed consent was obtained from all the participants.

## Supporting information

Supporting information.

Supporting information.

Supporting information.

Supporting information.

Supporting information.

## Data Availability

The data that support the findings of this study are available on request from the corresponding author. The data are not publicly available due to privacy or ethical restrictions. To protect participants' privacy and confidentiality the generated and analysed datasets of the interviews are not publicly available. Datasets of the literature search are available from the corresponding author upon request.
