# Supplementary material for: Up‐to‐dateness of German patient versions of clinical practice guidelines and potential influencing factors: A mixed‐methods study
Source: Clin Public Health Guidel. 2025 Feb 3;2(2):e70017. doi: 10.1002/gin2.70017 (PMC13563345; doi:10.1002/gin2.70017)
Supplement: Supplementary file 1 [file GIN2-2-e70017-s001.docx]

## Additional file 3 – Category system

| Content | Category | Subcodes | Rules of coding | Category specification |
| --- | --- | --- | --- | --- |
| Personal information/ characteristics | Gender | Female | One word |  |
|  |  | Male |  |  |
|  | Age | 30-39 | Number [y] | Years |
|  |  | 40-49 |  |  |
|  |  | 50-59 |  |  |
|  |  | 60-69 |  |  |
|  | Professional position | Methodological | One word | Experts’ current occupation |
|  |  | Clinical |  |  |
|  | Time in professional position | <1 | Number or Word [y] | Years |
|  |  | 1-5 |  |  |
|  |  | 6-10 |  |  |
|  |  | 11-15 |  |  |
|  | Tasks in PVG development | Development of general methodology/templates | Context |  |
|  |  | Coordination of PVG development |  |  |
|  |  | Organisation of patient involvement |  |  |
|  |  | Drafting of PVG |  |  |
|  |  | Drafting of PVG methods report |  | Experts’ participation in the development of methods reports for individual PVGs |
|  |  | Review of PVG |  |  |
|  |  | Dissemination |  |  |
|  | Number of accompanied PVGs | 1-5 | Number or Word | Number of PVGs in whose development experts were involved, at minimum 1 PVG, since experts were identified through author lists of PVGs |
|  |  | 6-10 |  |  |
|  |  | 31-35 |  |  |
|  | Guideline program | National Program for Disease Management Guidelines (NVL) | Word | Guideline programs in which experts were involved during PVG development |
|  |  | German Guideline Program in Oncology (GGPO) |  |  |
|  | Years of experience in PVG development | <1 | Number or Word [y] | Years |
|  |  | 1-5 |  |  |
|  |  | 6-10 |  |  |
|  |  | 11-15 |  |  |
| Development process of PVGs | Development process | Structured process | Context | Descriptions of the PVG development as a formal/ structured process |
|  |  | Shortened process during updating |  | Descriptions of shortened procedures during the updating of PVGs in comparison to the regular updating process |
|  |  | Duration of development process |  |  |
|  |  | Start time of development process |  | Descriptions of the starting point of the PVG development process, such as during the consultation phase or after finalisation of CPG |
|  |  | Editorial board |  | Descriptions on the acquisition/composition of the editorial board |
|  |  | Kick-off meeting |  |  |
|  |  | Conflicts of interest |  |  |
|  |  | Determining PVG content |  | Descriptions on the determination of the content and structure of a PVG |
|  |  | Development of draft |  |  |
|  |  | Review process   - Internal review process - Public consultation - Additional review |  |  |
|  |  | Dissemination |  |  |
|  | Influence factors | Extensive CPG | Context |  |
|  |  | Quality of CPG |  | Descriptions on the influence of methodological/reporting quality of CPGs on the development of PVGs |
|  |  | Patient representatives |  |  |
|  |  | Editorial Board   - Expertise on the subject - Opposing perceptions - Conflicts of interest - Exchange between members - Invariable pool of members - Availability of members |  | Descriptions on the influence of the editorial board on the development of PVGs |
|  |  | Templates |  |  |
|  |  | Review process |  |  |
| Up-to-dateness of PVGs | Up-to-dateness | General impression | Context |  |
|  |  | Exceptions in up-to-dateness   - Depending on chapter - Depending on subject - Other |  | The judgement regarding the up-to-dateness depends on the topics and chapters of PVGs |
|  | Influence factors | Scientific progress | Context |  |
|  |  | Print version of PVG |  |  |
|  |  | Review process of PVG |  |  |
|  |  | Time of planning of PVG |  |  |
|  |  | Up-to-dateness/updating of CPG |  | Experts’ descriptions of the up-to-dateness or updating of CPGs as an influence factor on the up-to-dateness of PVGs |
|  |  | Time/personnel resources |  |  |
|  | Suggestions for improvement | Earlier onset of PVG development | Context |  |
|  |  | Management of PVG development |  | Suggestions regarding the management of PVG development, such as defining clear responsibilities, timelines and milestones |
|  |  | Editorial board |  | Suggestions regarding the editorial board, such as time resources of members or responsibilities |
|  |  | Dynamic processes |  | Suggestions regarding dynamic processes, such as amendments or living guidelines |
|  |  | Availability of resources |  |  |
|  |  | Digitalisation |  |  |
