# Supplementary material for: Up‐to‐dateness of German patient versions of clinical practice guidelines and potential influencing factors: A mixed‐methods study
Source: Clin Public Health Guidel. 2025 Feb 3;2(2):e70017. doi: 10.1002/gin2.70017 (PMC13563345; doi:10.1002/gin2.70017)
Supplement: Supplementary file 2 [file GIN2-2-e70017-s002.docx]

**Additional file 5: Interview statements**

Development process of PVGs

ID06: *“Well, a patient guideline [PVG] can only be as good as what is written in the guideline [CPG]. And if there are no clear recommendations in it, which are well documented, this makes everything a bit more difficult.”*

Up-to-dateness of PVGs

*ID01: “Well, this [the up-to-dateness] depends on the field, as I said before. In oncology, for example, there is more happening in terms of treatment options compared to other areas. Therefore, this is probably quite different.”*

*ID06: “The only thing that is really important in terms of facilitating factors is that you also need experts, doctors, who are somehow trained in patient information. The same applies to patient representatives, who should also have a basic knowledge of patient information […].”*

*ID02: “Of course, the whole development process with printing is admittedly cumbersome. That is simply the nature of things and you cannot react quickly.”*

Suggestions for improvement

ID04: *“And I think it is very important to also give younger colleagues the opportunity to be involved and to contribute [to the PVG development], who perhaps simply do not have so much work as professors who are usually involved, so to speak.”*

ID05: *“Training programs for patient representatives are good, and perhaps we should also think about offering a compensation to the patient representatives who are involved.”*

*ID04: “I believe it is always useful to ensure communication and to develop clear timetables and certain milestones. For example, by defining which milestone should be reached at what time.”*

ID04: *“But if S3 guidelines [CPGs] would be updated more quickly or within the framework of living guidelines, and if the patient version [PVG] would be linked to this, that would be the quickest way, I think.”*

ID02: *“The most important aspect would be the digitalisation, to support the development process [of PVGs] by online tools, as we started with the guidelines [the CPGs], through online portals, i.e., content management systems. This means that the content is web-based, enabling a faster editing and especially a faster development of digital end products.”*
