# Supplementary material for: Up‐to‐dateness of German patient versions of clinical practice guidelines and potential influencing factors: A mixed‐methods study
Source: Clin Public Health Guidel. 2025 Feb 3;2(2):e70017. doi: 10.1002/gin2.70017 (PMC13563345; doi:10.1002/gin2.70017)
Supplement: Supplementary file 3 [file GIN2-2-e70017-s003.docx]

## Additional file 2 – Interview guide

The original German version of the interview guide has been translated with DeepL (https://www.deepl.com/translator).

**Up-to-dateness of patient versions of guidelines - Interview guide for experts**

*Presentation/Introduction*

Good afternoon. My name is Irma Hellbrecht from the Institute for Research in Operative Medicine (IFOM) at Witten/Herdecke University in Cologne. I am very pleased that you are supporting the study "Up-to-dateness of patient versions of clinical practice guidelines and potential influencing factors: a mixed-methods analysis" by participating in this interview.

I had already informed you in a written form about the purpose of this study. I will now ask you some questions, which I would like you to answer freely. There are no right or wrong answers, I simply want to hear your opinion or experience on various topics. Please get in touch at any time if you have not understood a question in terms of content or tone. Do you have any questions about this in advance? Do you agree that I record the interview on a recorder? This will help me with the subsequent transcription and evaluation.

As you have already read in the data protection statement, your data will be used exclusively for this current project and only in pseudonymised form. This means that a code will be assigned to your name and the data collected (age, number of accompanied patient versions of guidelines and years of experience) will be analysed in aggregated form and later presented in categories. The interview results are therefore published without any possibility of assigning them to your person, and specific information such as the names of specialist societies are made unrecognisable during transcription. The audio recording and transcription of the interview, as well as your declaration of consent, will be stored for a maximum of 10 years after the end of the study and will then be deleted. Do you agree with this?

Ok, then we can start. (Switch on the recording device!)

*General information about the person*

To compare the interviews and statements of the different participants, I would first like to ask you some general questions about your person.

- First of all, I note that you are female/male, is that correct?
- Would you please tell me your age?
- Are you currently working in a methodological or clinical position?
- What function do you perform in this [methodological/clinical] position? Thus, what are your tasks within this position?
- For how long have you been working in this position?
- How have you been involved in the development of patient versions of guidelines so far? Thus, what was your task during the development process?
- In how many patient versions of guidelines have you been involved during the development process?
- And in which program were these patient versions of guidelines mainly developed? I am referring, for example, to the German Guideline Program in Oncology or the program of National Disease Management Guidelines.
- How many years of experience do you have in developing patient versions of guidelines?

*Development process of patient versions of guidelines*

In the following, I would like to start by asking you some questions about the development process of patient versions of guidelines.

- Would you first briefly describe how the process of developing patient versions of guidelines takes place in your experience?

(If not already mentioned)

- What similarities/differences exist in the development of patient versions of guidelines compared to the development of S3 guidelines with regard to the time frame?
- Can you briefly describe the tasks of the editorial board in the development process?
- How are patient representatives involved in the development process?
- How is the external review carried out during the consultation phase?
- Which factors do you think can have a positive/negative influence on the development process of patient versions of guidelines?

(If not already mentioned)

- What influence do you think time resources have on the development process?
- What influence do you think personnel resources have on the development process?
- What influence do you think financial resources have on the development process?

*Up-to-dateness of patient versions of guidelines*

Now that we have talked about the process of developing patient versions of guidelines, I would like to talk with you about the up-to-dateness of patient versions of guidelines and also refer to the aspects we have just mentioned.

- How do you judge the up-to-dateness of patient versions of guidelines in Germany?

(If not already mentioned)

- For what reason do you consider that patient versions of guidelines are current/outdated?

IF CURRENT

- Now please think of patient versions of guidelines on rapidly evolving topics such as cancer. How would you judge the up-to-dateness of these patient versions of guidelines?
- In your experience, what factors can generally influence the up-to-dateness of patient versions of guidelines?

(If not already mentioned)

- What influence do you think the appointment of the editorial board has on the development process?
- What influence do you think the involvement of patient representatives has on the development process?
- What influence do you think does the involvement of experience in the patient version of guidelines has on the development process?
- What influence do you think does the target group of a patient version of guidelines has on the development process?
- What influence do you think does the external review during the consultation phase has on the development process?
- In your opinion, what influence does the additional development of a methods report for the patient version of guidelines have on the development process?
- Do you know of patient versions of guidelines from other programs or at the international level where a better up-to-dateness of patient versions of guidelines is ensured?

(If not already mentioned)

- Are there aspects that you would like to adapt for your development process?
- In your experience, what framework conditions should be established to ensure the up-to-dateness of patient versions of guidelines?

(If not yet mentioned)

- Do you have any concrete suggestions or ideas on how to improve the up-to-dateness of patient versions of guidelines in Germany?

As we have already reached the end of the interview, I would like to thank you very much for your participation and the time you have taken.
