# Supplementary material for: Up‐to‐dateness of German patient versions of clinical practice guidelines and potential influencing factors: A mixed‐methods study
Source: Clin Public Health Guidel. 2025 Feb 3;2(2):e70017. doi: 10.1002/gin2.70017 (PMC13563345; doi:10.1002/gin2.70017)
Supplement: Supplementary file 4 [file GIN2-2-e70017-s004.docx]

## Additional file 1 – Proposal for Master’s Thesis

University of Cologne

Faculty of Management, Economics and Social Sciences

Institute for Health Economics and Clinical Epidemiology

Proposal for Master Thesis

Timeliness of patient versions of clinical practice guidelines and potential methodological influencing factors: a mixed-methods analysis of patient versions of guidelines.

Submitted by:

Irma Hellbrecht

Health Economics B.Sc.

Date: 30.09.2022

**Introduction**

Clinical practice guidelines (CPG) aim to promote decision making of health professionals and patients, by providing explicit recommendations. They have the potential to optimize patient care and to reduce inadequate variation in practice [1]. Since patient and public awareness of CPGs is low [2] and CPGs are primarily tailored to healthcare providers, many guideline organizations also produce patient versions of CPG (PVoG) [3]. PVoGs go along with the corresponding CPGs and are therefore based on systematic research and on the current state of knowledge [4]. The up-to-dateness is not only a quality attribute to CPGs [4] but is also essential, since outdated recommendations can potentially lead to harmful, ineffective or suboptimal practices [5]. In 2001, an analysis by Shekelle et al. revealed that more than three-quarter of analysed CPGs published by the Agency for Healthcare Research and Quality were outdated and needed revision [6]. Accordingly, the timeliness of PVoGs particularly depends on the timeliness of CPGs. For this reason, efficient development processes of PVoGs and the exploration of further potential methodological influencing factors on the timeliness of PVoGs are desirable. At present, data about the timeliness of PVoGs in Germany and corresponding potential methodological influencing factors are not available. Therefore, the main objective of this Master Thesis is to perform a mixed-methods analysis on the timeliness of national PVoGs and the potential methodological influence factors.

**Methodology**

The mixed-methods analysis will comprise two main modules and will be based on a literature search and on expert interviews.

In module 1, the timeliness of PVoGs and potential methodological influence factors will be analysed based on a literature search. PVoGs will be included into this analysis, if they fulfil the following criteria: published in German, free accessible, defined as PVoG by authors, explicit link to corresponding CPG, current final version, date of research strategy of CPG available and publication date of PVoG available. To identify PVoGs on national level, the websites of the following organizations will be searched by one reviewer: Nationale Versorgungsleitlinien (NVL), Arbeitsgemeinschaft der Wissenschaftlichen Medizinischen Fachgesellschaften e.V. (AWMF), Leitlinienprogramm Onkologie (OL). Additionally, a manual search will be conducted in google and the reference lists of identified PVoGs will be screened. Title and abstract screening will be carried out by two independent reviewers and the full-texts of potentially relevant papers will be checked. The publication dates of the PVoGs will be compared with the time when the search strategies were performed in the CPGs to analyse the timeliness of PVoGs. The PVoGs and the CPGs will be screened and if available, corresponding methods papers will also be checked. Data will be extracted by one reviewer and verified by another. The following general information will be included into the a priori developed and piloted data extraction sheet: source, medical condition, publication date of PVoG and time when the search strategies were performed in the CPG. Information such as the number of pages, target audience, use of glossary, use of charts/illustrations, use of grade of recommendation, patient involvement, availability of methods paper, chronological order of the developing process/publication of CPG and PVoG (CPG first or CPG and PVoG in parallel) and whether the PVoG is available as online document, print or both will be extracted. The list of pre-specified methodological influencing factors may be extended during data extraction. The extracted information will be synthesized narratively and the timeliness of PVoGs will be reported by mean (standard deviation) and median (Interquartile range).

In module 2, further potential methodological influence factors will be explored by conducting interviews with experts in the development of PVoGs. Members of the Development Groups of PVoGs, previously identified within module 1 will be contacted via e-mail to determine 5-10 interview participants. An interview guide will be conducted a priori and piloted before interviewing. The interviews will be conducted via phone and will be recorded and transcribed by one reviewer. The analysis will be based on the qualitative content analysis according to Mayring [7], by applying the qualitative data analysis software MAXQDA.

**References**

1.	Graham R, Mancher M, Miller Wolman D, Greenfield S, Steinberg E. Clinical Practice Guidelines We Can Trust. Clinical Practice Guidelines We Can Trust, ed. R. Graham, et al. Washington (DC); 2011

2.	Loudon K, Santesso N, Callaghan M, Thornton J, Harbour J, Graham K, et al. Patient and public attitudes to and awareness of clinical practice guidelines: a systematic review with thematic and narrative syntheses. BMC Health Serv Res. 2014; 14: 321

3.	Santesso N, Morgano GP, Jack SM, Haynes RB, Hill S, Treweek S, et al. Dissemination of Clinical Practice Guidelines: A Content Analysis of Patient Versions. Med Decis Making. 2016; 36: 692-702

4.	Ärztliches Zentrum für Qualität in der Medizin (ÄZQ), Office des Leitlinienprogramms Onkologie (OL), Wissensmanagement A-IfM, (AWMF-IMWi). Erstellung von Patientenleitlinien zu S3-Leitlinien/NVL im Rahmen der Leitlinienprogramme. 2019

5.	Woolf SH, Grol R, Hutchinson A, Eccles M, Grimshaw J. Clinical guidelines: potential benefits, limitations, and harms of clinical guidelines. Bmj. 1999; 318: 527-30

6.	Shekelle PG, Ortiz E, Rhodes S, Morton SC, Eccles MP, Grimshaw JM, et al. Validity of the Agency for Healthcare Research and Quality clinical practice guidelines: how quickly do guidelines become outdated? Jama. 2001; 286: 1461-7

7.	Mayring P. Qualitative Inhaltsanalyse. Grundlagen und Techniken. 13 Aufl. Weinheim/Basel: Beltz; 2022
