# Supplementary material for: Up‐to‐dateness of German patient versions of clinical practice guidelines and potential influencing factors: A mixed‐methods study
Source: Clin Public Health Guidel. 2025 Feb 3;2(2):e70017. doi: 10.1002/gin2.70017 (PMC13563345; doi:10.1002/gin2.70017)
Supplement: Supplementary file 5 [file GIN2-2-e70017-s005.docx]

**Additional file 4 – List of excluded documents**

|  |  | **Item*** | **Reason for exclusion** |
| --- | --- | --- | --- |
| 1 |  | Association of the Scientific Medical Societies (German: AWMF). Breast cancer II - Advanced disease, recurrence and metastasis. [Brustkrebs II - Die fortgeschrittene Erkrankung, Rezidiv und Metastasierung.] 2011. Available from: https://www.awmf.org/uploads/tx_szleitlinien/032-045-OL-p3_S3_Brustkrebs_2011-08-abgelaufen.pdf. [Last accessed: 01.03.2023]. | Later version available |
| 2 |  | Association of the Scientific Medical Societies (German: AWMF). DEGAM Patient Information: Elevated TSH level - suspected hypothyroidism. [DEGAM Patienteninformation: Erhöhter TSH-Wert – Verdacht auf Schilddrüsenunterfunktion.] 2016. Available from: https://register.awmf.org/assets/guidelines/053-046p_S2k_erhoehter_TSH_Wert_2017-04-abgelaufen.pdf. [Last accessed: 01.03.2023]. | No PVG |
| 3 |  | Association of the Scientific Medical Societies (German: AWMF). DEGAM Patient Information: Gout. [DEGAM Patienteninformation: Gicht.] 2018. Available from: https://register.awmf.org/assets/guidelines/053-032p1_S3_Haeufige-Gichtanfaelle-chronische-Gicht_2020-02.pdf. [Last accessed: 01.03.2023]. | Duplicate |
| 4 |  | Association of the Scientific Medical Societies (German: AWMF). DEGAM Patient Information: Gout. [DEGAM Patienteninformation: Gicht.] 2018. Available from: https://www.awmf.org/uploads/tx_szleitlinien/053-032abp1_Gicht_2019-01_01.pdf. [Last accessed: 01.03.2023]. | No PVG |
| 5 |  | Association of the Scientific Medical Societies (German: AWMF). DEGAM Patient Information: Information on the Coronavirus Pandemic. [DEGAM Patienteninformation: Informationen zur Coronavirus-Pandemie.] 2021. Available from: https://www.awmf.org/uploads/tx_szleitlinien/053-054p_S1_Neues_CORONA_Virus_2021-11.pdf. [Last accessed: 01.03.2023]. | No PVG |
| 6 |  | Association of the Scientific Medical Societies (German: AWMF). DEGAM patient information: People with multiple diseases. [DEGAM Patienteninformation: Menschen mit mehreren Krankheiten.] 2017. Available from: https://register.awmf.org/assets/guidelines/053-047p_S3_Multimorbiditaet_2017-11.pdf. [Last accessed: 01.03.2023]. | No PVG |
| 7 |  | Association of the Scientific Medical Societies (German: AWMF). DEGAM Patient Information: Fatigue. [DEGAM Patienteninformation: Müdigkeit.] 2017. Available from: https://register.awmf.org/assets/guidelines/053-002p_S3_Muedigkeit_2020-02.pdf. [Last accessed: 01.03.2023]. | No PVG |
| 8 |  | Association of the Scientific Medical Societies (German: AWMF). DEGAM Patient Information: Nonvisible Hematuria (NSH). [DEGAM Patienteninformation: Nicht sichtbare Hämaturie (NSH).] 2020. Available from: https://www.awmf.org/fileadmin/user_upload/Leitlinien/053_D_Ges_fuer_Allgemeinmedizin_und_Familienmedizin/053-028pA4lang_S1_Nicht_sichtbare_Haematurie_2020-11.pdf. [Last accessed: 01.03.2023]. | No PVG |
| 9 |  | Association of the Scientific Medical Societies (German: AWMF). DEGAM patient information: family caregivers. [DEGAM Patienteninformation: Pflegende Angehörige.] 2018. Available from: https://register.awmf.org/assets/guidelines/053-006p2_S3_Pflegende-Angehoerige-von-Erwachsenen_2019-03_01.pdf. [Last accessed: 01.03.2023]. | No PVG |
| 10 |  | Association of the Scientific Medical Societies (German: AWMF). DEGAM Patient Information: Stroke Prevention, Recognition and Treatment. [DEGAM Patienteninformation: Schlaganfall vorbeugen, erkennen und behandeln.] 2020. Available from: https://register.awmf.org/assets/guidelines/053-011p_S3_Schlaganfall_2020-05.pdf. [Last accessed: 01.03.2023]. | No PVG |
| 11 |  | Association of the Scientific Medical Societies (German: AWMF). DEGAM patient information: Protection from overuse and underuse. [DEGAM Patienteninformation: Schutz vor Über- und Unterversorgung.] 2022. Available from: https://www.awmf.org/uploads/tx_szleitlinien/053-045p_S3_LG_Schutz_vor_Ueber_und_Unterversorgung_2022-04.pdf. [Last accessed: 01.03.2023]. | Living Guideline |
| 12 |  | Association of the Scientific Medical Societies (German: AWMF). Diagnosis and treatment of eating disorders. Patient guideline. [Diagnostik und Behandlung von Essstörungen. Patientenleitlinie.] 2015. Available from: https://www.awmf.org/uploads/tx_szleitlinien/051-026p_Essstoerungen_2015-06_01.pdf. [Last accessed: 01.03.2023]. | CPG newer than PVG |
| 13 |  | Association of the Scientific Medical Societies (German: AWMF). A "Long-/Post-COVID Syndrome" guideline for affected individuals, family members, related persons and caregivers, based on a physician's guideline. [Eine Leitlinie “Long-/Post-COVID-Syndrom" für Betroffene, Angehörige, nahestehende und pflegende Personen, die sich auf eine ärztliche Leitlinie stützt.] 2021. Available from: https://www.awmf.org/uploads/tx_szleitlinien/020-027p_S1_Post_COVID_Long_COVID_2021-12.pdf. [Last accessed: 01.03.2023]. | Living Guideline |
| 14 |  | Association of the Scientific Medical Societies (German: AWMF). Early detection of breast cancer. A decision aid for women. [Früherkennung von Brustkrebs. Eine Entscheidungshilfe für Frauen.] 2010. Available from: https://www.awmf.org/uploads/tx_szleitlinien/032-045OLp1_S3_Brustkrebs_Fueherkennung_2010-abgelaufen.pdf. [Last accessed: 01.03.2023]. | Later version available |
| 15 |  | Association of the Scientific Medical Societies (German: AWMF). Premature birth - What you should know as expectant parents. Initial information on a complex topic. [Frühgeburt - Was Sie als werdende Eltern wissen sollten. Erste Informationen zu einem komplexen Thema.] 2022. Available from: https://www.awmf.org/uploads/tx_szleitlinien/015-025p_S2k_Praevention-Therapie_Fruehgeburt_2022-08.pdf. [Last accessed: 01.03.2023]. | No research strategy available |
| 16 |  | Association of the Scientific Medical Societies (German: AWMF). Understanding and managing functional body complaints. A guideline for affected people and their families. Long version. [Funktionelle Körperbeschwerden verstehen und bewältigen. Eine Leitlinie für Betroffene und ihre Angehörigen. Langfassung.] 2018. Available from: https://www.awmf.org/uploads/tx_szleitlinien/051-001p1_S3_Funktionelle_Koerperbeschwerden_2020-01.pdf. [Last accessed: 01.03.2023]. | Ambiguous research dates |
| 17 |  | Association of the Scientific Medical Societies (German: AWMF). Health information: fissure and pit sealing. [Gesundheitsinformation: Fissuren- und Grübchenversiegelung.] 2018. Available from: https://www.awmf.org/uploads/tx_szleitlinien/083-002p_S3_Fissuren-Gruebchenversiegelung_2019-01-abgelaufen.pdf. [Last accessed: 01.03.2023]. | No PVG |
| 18 |  | Association of the Scientific Medical Societies (German: AWMF). Health guideline: Early Detection of Colorectal Cancer. [Gesundheitsleilinie: Früherkennung von Darmkrebs.] 2015. Available from: https://www.awmf.org/uploads/tx_szleitlinien/021-007OLp1_S3_KRK_2018-04_01.pdf. [Last accessed: 01.03.2023]. | Version of CPG/CPG report unclear |
| 19 |  | Association of the Scientific Medical Societies (German: AWMF). Information for patients on the AWMF guideline: Diagnosis and treatment of hormone-inactive pituitary tumors. [Information für Patient*innen zur AWMF-Leitlinie: Diagnostik und Behandlung von hormoninaktiven Hypophysentumoren.] 2019. Available from: https://www.awmf.org/uploads/tx_szleitlinien/089-002p_S2k_Diagnostik-Therapie-hormoninaktiver-Hypophsenaddenome_2020-04.pdf. [Last accessed: 01.03.2023]. | No research strategy available |
| 20 |  | Association of the Scientific Medical Societies (German: AWMF). Guideline Multiple Sclerosis for Patients. [Leitlinie Multiple Sklerose für Patientinnen und Patienten.] 2022. Available from: https://www.awmf.org/uploads/tx_szleitlinien/030-050p_S2e_Multiplen-Sklerose_2022-05.pdf. [Last accessed: 01.03.2023]. | No research strategy available |
| 21 |  | Association of the Scientific Medical Societies (German: AWMF). Patient version: S3 guideline Sinus pilonidalis (coccygeal fistula). [Patientenfassung: S3-Leitlinie Sinus pilonidalis (Steißbeinfistel).] 2020. Available from: https://www.awmf.org/uploads/tx_szleitlinien/081-009p_S3_Sinus_pilonidalis_2020-07.pdf. [Last accessed: 01.03.2023]. | Publication year/month not available |
| 22 |  | Association of the Scientific Medical Societies (German: AWMF). Patient Information: Interstitial Cystitis IC/BPS. [Patienteninformation: Interstitielle Cystitis IC/BPS.] 2018. Available from: https://www.awmf.org/uploads/tx_szleitlinien/043-050p_S2k_Diagnostik_Therapie_Interstitielle_Cystitis_2018-10.pdf. [Last accessed: 01.03.2023]. | No research strategy available |
| 23 |  | Association of the Scientific Medical Societies (German: AWMF). Patient Information: Cubital Tunnel Syndrome - Diagnosis and Therapy. [Patienteninformation Kubitaltunnelsyndrom– Diagnostik und Therapie.] 2017. Available from: https://register.awmf.org/assets/guidelines/005-009p_S3_Kubitaltunnelsyndrom-Diagnostik-Therapie_2018-03.pdf. [Last accessed: 01.03.2023]. | No PVG |
| 24 |  | Association of the Scientific Medical Societies (German: AWMF). Patient information microcephaly. [Patienteninformation Mikrozephalie.] Available from: https://www.awmf.org/uploads/tx_szleitlinien/022-028p_S2k_Klassifikation_Diagnostik_Mikrozephalie_2019-11.pdf. [Last accessed: 01.03.2023]. | No PVG |
| 25 |  | Association of the Scientific Medical Societies (German: AWMF). Patient information: Sleepless nights - Why? What helps? [Patienteninformation Schlaflose Nächte - Warum? Was hilft?] 2018. Available from: https://www.awmf.org/uploads/tx_szleitlinien/063-003p2_S3_Insomnie-Erwachsene_2018-02-verlaengert.pdf. [Last accessed: 01.03.2023]. | No PVG |
| 26 |  | Association of the Scientific Medical Societies (German: AWMF). Patient information on the guideline "Caries prophylaxis in permanent teeth: Basic recommendations". [Patienteninformation zur Leitlinie „Kariesprophylaxe bei bleibenden Zähnen: Grundlegende Empfehlungen”.] 2016. Available from: https://register.awmf.org/assets/guidelines/083-021p_S2k_Kariesprophylaxe_2017-12.pdf. [Last accessed: 01.03.2023]. | No PVG |
| 27 |  | Association of the Scientific Medical Societies (German: AWMF). Early stage colorectal cancer patient guideline. [Patientenleilinie Darmkrebs im frühen Stadium.] 2019. Available from: https://www.awmf.org/uploads/tx_szleitlinien/021-007OLp2_S3_KRK_2020-08_01.pdf. [Last accessed: 01.03.2023]. | Duplicate |
| 28 |  | Association of the Scientific Medical Societies (German: AWMF). Patient guideline on advanced colorectal cancer. A guide for patients. [Patientenleitline Darmkrebs im fortgeschrittenen Stadium. Ein Ratgeber für Patientinnen und Patienten.] 2014. Available from: https://www.awmf.org/uploads/tx_szleitlinien/021-007OLp3_S3_KRK_2018-04_01.pdf. [Last accessed: 01.03.2023]. | Later version available |
| 29 |  | Association of the Scientific Medical Societies (German: AWMF). Patient guideline on early stage breast cancer. [Patientenleitlinie Brustkrebs im frühen Stadium.] 2018. Available from: https://www.awmf.org/uploads/tx_szleitlinien/032-045OLp2_S3_Mammakarzinom_2019-04.pdf. [Last accessed: 01.03.2023]. | Duplicate |
| 30 |  | Association of the Scientific Medical Societies (German: AWMF). Chronic Lymphocytic Leukemia (CLL) Patient Guideline. [Patientenleitlinie Chronische lymphatische Leukämie (CLL).] 2018. Available from: https://www.awmf.org/uploads/tx_szleitlinien/018-032OLp_S3_Chronisch-lymphatische-Leukaemie_2021-07.pdf. [Last accessed: 01.03.2023]. | Duplicate |
| 31 |  | Association of the Scientific Medical Societies (German: AWMF). Patient guideline cervical cancer. A guideline for patients. [Patientenleitlinie Gebärmutterhalskrebs. Eine Leitlinie für Patientinnen.] 2015. Available from: https://www.awmf.org/uploads/tx_szleitlinien/032-033OLp_S3_Gebaermutterhalskrebs_Zervixkarzinom_2020-08.pdf. [Last accessed: 01.03.2023]. | Later version available |
| 32 |  | Association of the Scientific Medical Societies (German: AWMF). [Patient Guideline Testicular Cancer. A guideline for the investigation, treatment and follow-up of testicular cancer (preliminary version 0.2).] Patientenleitlinie Hodenkrebs. Eine Leitlinie zur Untersuchung, Behandlung und Nachsorge des Hodenkrebses (vorläufige Version 0.2). 2021. Available from: https://www.awmf.org/uploads/tx_szleitlinien/043-049OLp_S3_Hodenkebs_2022-09-vorlaeufig_01.pdf. [Last accessed: 01.03.2023]. | Duplicate |
| 33 |  | Association of the Scientific Medical Societies (German: AWMF). Hodgkin lymphoma patient guideline. [Patientenleitlinie Hodgkin Lymphom.] 2022. Available from: https://www.awmf.org/uploads/tx_szleitlinien/018-029OLp_S3_Hodgkin-Lymphom_Erwachsene_Diagnostik_Therapie_Nachsorge_2022-04.pdf. [Last accessed: 01.03.2023]. | Duplicate |
| 34 |  | Association of the Scientific Medical Societies (German: AWMF). Patient guideline: Cutaneous malignant lymphomas. A guideline for examination, treatment and follow-up. [Patientenleitlinie: Kutane maligne Lymphome. Eine Leitlinie zur Untersuchung, Behandlung und Nachsorge.] 2021. Available from: https://register.awmf.org/assets/guidelines/032-027p_S2k_Kutane_Lymphome_2021-09_01.pdf. [Last accessed: 01.03.2023]. | No research strategy available |
| 35 |  | Association of the Scientific Medical Societies (German: AWMF). Patient Guideline Melanoma. A guideline for the examination, treatment and follow-up of black skin cancer. [Patientenleitlinie Melanom. Eine Leitlinie zur Untersuchung, Behandlung und Nachsorge des schwarzen Hautkrebses.] 2019. Available from: https://www.awmf.org/uploads/tx_szleitlinien/032-024OLp_S3_Melanom-Diagnostik-Therapie-Nachsorge_2020-08_1.pdf. [Last accessed: 01.03.2023]. | Duplicate |
| 36 |  | Association of the Scientific Medical Societies (German: AWMF). Patient guideline oral cavity cancer. A guide for patients. [Patientenleitlinie Mundhöhlenkrebs. Ein Ratgeber für Patientinnen und Patienten.] 2014. Available from: https://www.awmf.org/uploads/tx_szleitlinien/007-100p_S3_Mundhoehlenkarzinom_2020-08.pdf. [Last accessed: 01.03.2023]. | Duplicate |
| 37 |  | Association of the Scientific Medical Societies (German: AWMF). Patient Guideline Prostate Cancer I - Localized Prostate Carcinoma. An evidence-based patient guide to the S3 guideline Early detection, diagnosis, and treatment of the various stages of prostate cancer. 3rd ed. [Patientenleitlinie Prostatakrebs I – Lokal begrenztes Prostatakarzinom. Ein evidenzbasierter Patientenratgeber zur S3-Leitlinie Früherkennung, Diagnose und Therapie der verschiedenen Stadien des Prostatakarzinoms. 3. Auflage.] 2018. Available from: https://www.awmf.org/uploads/tx_szleitlinien/043-022OLp2_S3_Prostatakarzinom_2021-07_01.pdf. [Last accessed: 01.03.2023]. | Duplicate |
| 38 |  | Association of the Scientific Medical Societies (German: AWMF). Patient Guideline Prostate Cancer II - Locally Advanced and Metastatic Prostate Cancer. An evidence-based patient guide to the S3 guideline Early detection, diagnosis, and treatment of the various stages of prostate cancer. 4th ed. [Patientenleitlinie Prostatakrebs II – Lokal fortgeschrittenes und metastasiertes Prostatakarzinom. Ein evidenzbasierter Patientenratgeber zur S3-Leitlinie Früherkennung, Diagnose und Therapie der verschiedenen Stadien des Prostatakarzinoms. 4. Auflage.] 2018. Available from: https://www.awmf.org/uploads/tx_szleitlinien/043-022OLp3_S3_Prostatakarzinom_2021-07.pdf. [Last accessed: 01.03.2023]. | Duplicate |
| 39 |  | Association of the Scientific Medical Societies (German: AWMF). Patient Guideline Psychooncology. Psychosocial support for cancer patients and relatives. [Patientenleitlinie Psychoonkologie. Psychosoziale Unterstützung für Krebspatienten und Angehörige.] 2016. Available from: https://www.awmf.org/uploads/tx_szleitlinien/032-051OLp_S3_Psychoonkologie_2018-08.pdf. [Last accessed: 01.03.2023]. | Duplicate |
| 40 |  | Association of the Scientific Medical Societies (German: AWMF). Patient Guideline: Supportive Therapy. Prevention and treatment of side effects of cancer treatment. [Patientenleitlinie Supportive Therapie. Vorbeugung und Behandlung von Nebenwirkungen einer Krebsbehandlung.] 2018. Available from: https://www.awmf.org/uploads/tx_szleitlinien/032-054OLp_S3_Supportiv_2018-04-abgelaufen.pdf. [Last accessed: 01.03.2023]. | Duplicate |
| 41 |  | Association of the Scientific Medical Societies (German: AWMF). Patient guideline for the National Health Care Guideline: Asthma. [Patientenleitlinie zur Nationalen VersorgungsLeitlinie: Asthma.] 2021. Available from: https://www.awmf.org/uploads/tx_szleitlinien/nvl-002p_S3_Asthma_2022-06.pdf. [Last accessed: 01.03.2023]. | Duplicate |
| 42 |  | Association of the Scientific Medical Societies (German: AWMF). Patient Guideline for the National Health Care Guideline: Heart Failure. [Patientenleitlinie zur Nationalen VersorgungsLeitlinie: Herzschwäche.] 2020. Available from: https://www.awmf.org/uploads/tx_szleitlinien/nvl-006pll_S3_Chronische_Herzinsuffizienz_2022-07.pdf. [Last accessed: 01.03.2023]. | Duplicate |
| 43 |  | Association of the Scientific Medical Societies (German: AWMF). Patient Guideline for the National Health Care Guideline: Low Back Pain. [Patientenleitlinie zur Nationalen VersorgungsLeitlinie: Kreuzschmerz.] 2017. Available from: https://www.awmf.org/uploads/tx_szleitlinien/nvl-007p_S3_Kreuzschmerz_2017-11-abgelaufen.pdf. [Last accessed: 01.03.2023]. | Duplicate |
| 44 |  | Association of the Scientific Medical Societies (German: AWMF). Patient Guideline for the National Health Care Guideline: Unipolar Depression. [Patientenleitlinie zur Nationalen VersorgungsLeitlinie: Unipolare Depression.] 2016. Available from: https://www.awmf.org/uploads/tx_szleitlinien/nvl-005p_S3_Unipolare_Depression_2022-10.pdf. [Last accessed: 01.03.2023]. | Duplicate |
| 45 |  | Association of the Scientific Medical Societies (German: AWMF). Patient version - Guideline: S2k-LL Indication knee arthroplasty. [Patientenversion - Leitlinie: S2k-LL Indikation Knieendoprothese.] 2018. Available from: https://www.awmf.org/uploads/tx_szleitlinien/033-052p_S2k_Knieendoprothese_2018-04_01.pdf. [Last accessed: 01.03.2023]. | No research strategy available |
| 46 |  | Association of the Scientific Medical Societies (German: AWMF). Patient Version "Medication Safety in Pediatric Emergencies." [Patientenversion „Medikamentensicherheit bei Kindernotfällen“.] 2021. Available from: https://www.awmf.org/uploads/tx_szleitlinien/027-071p_S2k_Medikamentensicherheit-bei.Kindernotf%C3%A4llen_2021-03.pdf. [Last accessed: 01.03.2023]. | No research strategy available |
| 47 |  | Association of the Scientific Medical Societies (German: AWMF). Patient version "Medication safety in pediatric anesthesia". [Patientenversion „Medikamentensicherheit in der Kinderanästhesie“.] 2021. Available from: https://www.awmf.org/uploads/tx_szleitlinien/001-033p_S2k_Medikamentensicherheit__Kinderan%C3%A4sthesie_20121-12.pdf. [Last accessed: 01.03.2023]. | No research strategy available |
| 48 |  | Association of the Scientific Medical Societies (German: AWMF). Patient version of the S3 guideline: Pain assessment in older people in full inpatient care for the elderly. [Patientenversion zur S3-Leitlinie: Schmerzassessment bei älteren Menschen in der vollstationären Altenhilfe.] Available from: https://www.awmf.org/uploads/tx_szleitlinien/145-001p_S3_Schmerzassessment-bei-aelteren-Menschen_in-der-vollstationaeren_Altenhilfe_2018-11_1-abgelaufen.pdf. [Last accessed: 01.03.2023]. | No PVG |
| 49 |  | Association of the Scientific Medical Societies (German: AWMF). Patient guideline cancer of the uterine body. [Patientinnenleitlinie Krebs des Gebärmutterkörpers.] 2021. Available from: https://www.awmf.org/uploads/tx_szleitlinien/032-034OLp_S3_Endometriumkarzinom-Diagnostik-Therpie-Nachsorge-Gebaermutterkoerperkrebs_2022-02_01.pdf. [Last accessed: 01.03.2023]. | Duplicate |
| 50 |  | Association of the Scientific Medical Societies (German: AWMF). S2k guideline: management of large vessel vasculitis - annotated patient version. [S2k Leitlinie: Management der Großgefäßvaskulitiden - Kommentierte Patientenfassung.] Available from: https://www.awmf.org/uploads/tx_szleitlinien/060-007p_S2k_Management_Gro%C3%9Fgef%C3%A4%C3%9Fvaskulitiden_2020-08.pdf. [Last accessed: 01.03.2023]. | No research strategy available |
| 51 |  | Association of the Scientific Medical Societies (German: AWMF). Mucous membrane pemphigoid. Information for patients. [Schleimhautpemphigoid. Informationen für Patientinnen und Patienten.] 2022. Available from: https://www.awmf.org/uploads/tx_szleitlinien/013-102p_S2k_Diagnostik-Therapie-Schleimhautpemphigoid_2022-07.pdf. [Last accessed: 01.03.2023]. | No PVG |
| 52 |  | Association of the Scientific Medical Societies (German: AWMF). Vulvar cancer: a guideline for patients. [Vulvakrebs: Eine Leitlinie für Patientinnen.] 2021. Available from: https://register.awmf.org/assets/guidelines/015-059p_S2k_Vulvakarzinom_und_Vorstufen_Diagnostik_Therapie_2016-10.pdf. [Last accessed: 01.03.2023]. | No research strategy available |
| 53 |  | German Medical Association (German: BÄK), National Association of Statutory Health Insurance Physicians (German: KBV), Association of the Scientific Medical Societies (German: AWMF). Patient Guideline for the National Health Care Guideline Chronic Coronary Heart Disease. 3rd ed. [Patientenleitlinie zur Nationalen VersorgungsLeitlinie Chronische Koronare Herzkrankheit: KHK. 3. Auflage.] 2019. Available from: https://www.patienten-information.de/medien/patientenleitlinien/khk-3aufl-vers1-pll.pdf. [Last accessed: 01.03.2023]. | Duplicate |
| 54 |  | German Medical Association (German: BÄK), National Association of Statutory Health Insurance Physicians (German: KBV), Association of the Scientific Medical Societies (German: AWMF). Patient Guideline for the National Health Care Guideline: Type 2 Diabetes Prevention and Treatment Strategies for Foot Complications. [Patientenleitlinie zur Nationalen VersorgungsLeitlinie: Typ-2-Diabetes Prävention und Behandlungsstrategien für Fußkomplikationen.] 2007. Available from: https://www.leitlinien.de/themen/diabetes/archiv/archiv-nvl-typ-2-diabetes-praeventions-und-behandlungsstrategien-fuer-fusskomplikationen?b_start:int=0. [Last accessed: 01.03.2023]. | No research strategy available |
| 55 |  | German Diabetes Association (German: DDG). Diabetes and pregnancy. Patient version of the guideline of the German Diabetes Association. [Diabetes und Schwangerschaft. Patientenversion der Leitlinie der Deutschen Diabetes-Gesellschaft.] 2008. Available from: https://www.diabetesde.org/system/files/documents/patientenleitlinie_diabetes_und_schwangerschaft.pdf. [Last accessed: 01.03.2023]. | No access to CPG/ CPG report |
| 56 |  | German Diabetes Association (German: DDG). Diabetes and pregnancy. Patient version of the guideline of the German Diabetes Association. [Diabetes und Schwangerschaft. Patientenversion der Leitlinie der Deutschen Diabetes-Gesellschaft.] 2008. Available from: https://www.sjk.de/sites/default/files/downloads/Patientenleitlinie_Diabetes_und_Schwangerschaft.pdf. [Last accessed: 01.03.2023]. | Duplicate |
| 57 |  | European Society for Medical Oncology (ESMO). Non-small-cell lung cancer (NSCLC). An ESMO guide for patients. Patient information based on ESMO Clinical Practice Guidelines. [Eine ESMO-Patientenleitlinie - Patienteninformation auf Grundlage der ESMO-Leitlinien für die klinische Praxis.] 2017. Available from: https://www.esmo.org/content/download/123909/2350201/file/DE-Nicht-Kleinzelliges-Bronchialkarzinom-(NSCLC)-Patientenleitlinie.pdf. [Last accessed: 01.03.2023]. | No research strategy available |
| 58 |  | European Association for the Study of the Liver (EASL). Primary biliary cholangitis (PBC). Living with the diagnosis. [Primär biliäre Cholangitis (PBC). Leben mit der Diagnose.] 2019. Available from: https://www.leberhilfe.org/wp-content/uploads/2019/05/PBC-Leitlinie_de_02-2019_web.pdf. [Last accessed: 01.03.2023]. | No access to CPG/ CPG report |
| 59 |  | German Guideline Program in Oncology (German: GGPO) of the Association of the Scientific Medical Societies (German: AWMF), the German Cancer Society (German: DKG), and the German Cancer Aid. Health guideline early detection of colorectal cancer. 1st edition. [Gesundheitsleitlinie Früherkennung von Darmkrebs 1. Auflage.] 2015. Available from: https://www.leitlinienprogramm-onkologie.de/fileadmin/user_upload/Downloads/Patientenleitlinien/Gesundheitsleitlinie_Darmkrebs_Frueherkennung.pdf. [Last accessed: 01.03.2023]. | Duplicate |
| 60 |  | German Guideline Program in Oncology (German: GGPO) of the Association of the Scientific Medical Societies (German: AWMF), the German Cancer Society (German: DKG), and the German Cancer Aid. Health guideline early detection of prostate cancer. Information for men. 2nd edition. [Gesundheitsleitlinie Früherkennung von Prostatakrebs. Information für Männer. 2. Auflage.] 2015. Available from: https://www.leitlinienprogramm-onkologie.de/fileadmin/user_upload/Downloads/Patientenleitlinien/Prostatakrebs/Patientenleitlinie_Prostatakrebs_Frueherkennung-1870075.pdf. [Last accessed: 01.03.2023]. | Version of CPG/CPG report unclear |
| 61 |  | German Guideline Program in Oncology (German: GGPO) of the Association of the Scientific Medical Societies (German: AWMF), the German Cancer Society (German: DKG), and the German Cancer Aid. Health guideline prevention of skin cancer. 1st edition. [Gesundheitsleitlinie Prävention von Hautkrebs. 1. Auflage.] 2016. Available from: https://www.leitlinienprogramm-onkologie.de/fileadmin/user_upload/Downloads/Patientenleitlinien/Gesundheitsleitlinie_Hautkrebspraevention-1700046.pdf. [Last accessed: 01.03.2023]. | Version of CPG/CPG report unclear |
| 62 |  | German Guideline Program in Oncology (German: GGPO) of the Association of the Scientific Medical Societies (German: AWMF), the German Cancer Society (German: DKG), and the German Cancer Aid. Patient Guideline Bladder Cancer. A guideline for patients. [Patientenleitlinie Blasenkrebs. Eine Leitlinie für Patientinnen und Patienten.] 2017. Available from: https://www.leitlinienprogramm-onkologie.de/fileadmin/user_upload/Downloads/Patientenleitlinien/Patientenleitlinie_Blasenkrebs_v3.pdf. [Last accessed: 01.03.2023]. | Version of CPG/CPG report unclear |
| 63 |  | German Guideline Program in Oncology (German: GGPO) of the Association of the Scientific Medical Societies (German: AWMF), the German Cancer Society (German: DKG), and the German Cancer Aid. Patient Guideline Testicular Cancer. A guideline for diagnosis, therapy and follow-up. [Patientenleitlinie Hodenkrebs. Eine Leitlinie zur Diagnostik, Therapie und Nachsorge.] 2021. Available from: https://www.leitlinienprogramm-onkologie.de/fileadmin/user_upload/Downloads/Patientenleitlinien/Patientenleitlinie_Hodenkrebs_1850012_PRAEFINAL.pdf. [Last accessed: 01.03.2023]. | Version of CPG/CPG report unclear |
| 64 |  | German Guideline Program in Oncology (German: GGPO) of the Association of the Scientific Medical Societies (German: AWMF), the German Cancer Society (German: DKG), and the German Cancer Aid. Hodgkin lymphoma patient guideline. [Patientenleitlinie Hodgkin Lymphom.] 2022. Available from: https://www.leitlinienprogramm-onkologie.de/fileadmin/user_upload/Downloads/Patientenleitlinien/Patientenleitlinie_Hodgkin-Lymphom-1890012.pdf. [Last accessed: 01.03.2023]. | Living Guideline |
| 65 |  | German Guideline Program in Oncology (German: GGPO) of the Association of the Scientific Medical Societies (German: AWMF), the German Cancer Society (German: DKG), and the German Cancer Aid. Patient Guideline Prostate Cancer I - Localized Prostate Carcinoma. An evidence-based patient guide to the S3 guideline Early detection, diagnosis, and treatment of the various stages of prostate cancer. 3rd ed. [Patientenleitlinie Prostatakrebs I – Lokal begrenztes Prostatakarzinom. Ein evidenzbasierter Patientenratgeber zur S3-Leitlinie Früherkennung, Diagnose und Therapie der verschiedenen Stadien des Prostatakarzinoms. 3. Auflage.] 2018. Available from: https://www.leitlinienprogramm-onkologie.de/fileadmin/user_upload/Downloads/Patientenleitlinien/Patientenleitlinie_Prostatakrebs_1-1830011.pdf. [Last accessed: 01.03.2023]. | Publication year/month not available |
| 66 |  | German Guideline Program in Oncology (German: GGPO) of the Association of the Scientific Medical Societies (German: AWMF), the German Cancer Society (German: DKG), and the German Cancer Aid. Patient Guideline Prostate Cancer II - Locally Advanced and Metastatic Prostate Cancer. An evidence-based patient guide to the S3 guideline Early detection, diagnosis and treatment of the different stages of prostate cancer. 4th edition. [Patientenleitlinie Prostatakrebs II – Lokal fortgeschrittenes und metastasiertes Prostatakarzinom. Ein evidenzbasierter Patientenratgeber zur S3-Leitlinie Früherkennung, Diagnose und Therapie der verschiedenen Stadien des Prostatakarzinoms 4. Auflage.] 2018. Available from: https://www.leitlinienprogramm-onkologie.de/fileadmin/user_upload/Downloads/Patientenleitlinien/Patientenleitlinie_Prostatakrebs_2-184-0011web.pdf. [Last accessed: 01.03.2023]. | Publication year/month not available |
| 67 |  | German Guideline Program in Oncology (German: GGPO) of the Association of the Scientific Medical Societies (German: AWMF), the German Cancer Society (German: DKG), and the German Cancer Aid. Patient Guideline Supportive Therapy. Prevention and treatment of side effects of cancer treatment. [Patientenleitlinie Supportive Therapie. Vorbeugung und Behandlung von Nebenwirkungen einer Krebsbehandlung.] 2018. Available from: https://www.leitlinienprogramm-onkologie.de/fileadmin/user_upload/Downloads/Patientenleitlinien/Patientenleitlinie_Supportive_Therapie-1740018.pdf. [Last accessed: 01.03.2023]. | Version of CPG/CPG report unclear |
| 68 |  | German Guideline Program in Oncology (German: GGPO) of the Association of the Scientific Medical Societies (German: AWMF), the German Cancer Society (German: DKG), and the German Cancer Aid. Patient guideline on early stage breast cancer. [Patientinnenleitlinie Brustkrebs im frühen Stadium.] 2018. Available from: https://www.leitlinienprogramm-onkologie.de/fileadmin/user_upload/Downloads/Patientenleitlinien/Patientenleitlinie_Brustkrebs_im_fruehen_Stadium_1820010.pdf. [Last accessed: 01.03.2023]. | Version of CPG/CPG report unclear |
| 69 |  | German Guideline Program in Oncology (German: GGPO) of the Association of the Scientific Medical Societies (German: AWMF), the German Cancer Society (German: DKG), and the German Cancer Aid. Patient guideline ovarian cancer. A guide for patients. [Patientinnenleitlinie Eierstockkrebs. Ein Ratgeber für Patientinnen.] 2018. Available from: https://www.leitlinienprogramm-onkologie.de/fileadmin/user_upload/Downloads/Patientenleitlinien/Patientenleitlinie_Eierstockkrebs-1920010.pdf. [Last accessed: 01.03.2023]. | Version of CPG/CPG report unclear |
| 70 |  | German Guideline Program in Oncology (German: GGPO) of the Association of the Scientific Medical Societies (German: AWMF), the German Cancer Society (German: DKG), and the German Cancer Aid. Patient guideline metastatic breast cancer. [Patientinnenleitlinie Metastasierter Brustkrebs.] 2018. Available from: https://www.leitlinienprogramm-onkologie.de/fileadmin/user_upload/Downloads/Patientenleitlinien/Patientenleitlinie_Brustkrebs_metastasiert.pdf. [Last accessed: 01.03.2023]. | Version of CPG/CPG report unclear |

*Translated with DeepL (https://www.deepl.com/translator)
